# Support for lowering cervical cancer screening age to 25 for women living with HIV: retrospective cross-sectional programmatic data from Botswana

**DOI:** 10.1186/s12905-022-01680-7

**Published:** 2022-04-02

**Authors:** Doreen Ramogola-Masire, Surbhi Grover, Anikie Mathoma, Barati Monare, Lesego Gabaitiri, Lisa Bazzett-Matabele, GJustus Hofmeyr, Chelsea Morroni, Rebecca Luckett

**Affiliations:** 1 Department of Obstetrics and Gynecology and Office of Research and Graduate Studies, University of Botswana, Corner of Notwane and Mabuto Road, Gaborone, Botswana; 2 Department of Radiation Oncology, University of Pennsylvania, Philadelphia, USA; 3 Botswana U-Penn Partnership, Gaborone, Botswana; 4 Office of Research, Faculty of Medicine, University of Botswana, Gaborone, Botswana; 5 Department of Statistics, Faculty of Social Sciences, University of Botswana, Gaborone, Botswana; 6 Botswana-Harvard AIDS Institute Partnership, Gaborone, Botswana; 7 MRC Centre for Reproductive Health, University of Edinburgh, Edinburgh, UK; 8 Department of Obstetrics and Gynecology, Beth Israel Deaconess Medical Center, Boston, MA USA

**Keywords:** Botswana, Cervical pre-cancer, Cervical cancer screening, Colposcopy, Cryotherapy, Loop electrical excision procedure, See-and-treat, Visual inspection after acetic acid

## Abstract

**Background:**

Women living with human immunodeficiency virus (HIV) tend to develop cervical cancer at a younger age than women without HIV. The World Health Organization’s (WHO) 2021 guidelines for screening and treatment of cervical pre-cancer lesions for cervical cancer prevention include a conditional recommendation for initiating screening at age 25 for women living with HIV (WLWH). This recommendation is based on low-certainty evidence, and WHO calls for additional data. We describe the association of age and HIV status with visual inspection with acetic acid (VIA) positivity and cervical intraepithelial neoplasia grade two or higher (CIN2+) in Botswana.

**Methods:**

This was a retrospective cross-sectional study of 5714 participants aged 25 to 49 years who underwent VIA screening in a clinic mainly serving WLWH. VIA-positive women received cryotherapy if eligible or were referred for colposcopy and excisional treatment. Known cervical cancer risk factors, screening outcome, and histological results were extracted from the program database. We compared the proportions and association of VIA positivity and CIN2+ by age and HIV status.

**Results:**

The median age was 35 years [IQR 31–39], and 18% of the women were aged 25–29. Ninety percent were WLWH; median CD4 count was 250 cells/µL [IQR 150–428], and 34.2% were on anti-retroviral treatment (ART). VIA-positivity was associated with younger age (OR 1.48, CI 1.28, 1.72 for 25–29 years vs. 30–49 years), and HIV-positivity (OR 1.85, CI 1.51, 2.28). CIN2+ was only associated with HIV-positivity (OR 6.12, CI 3.39, 11.10), and proportions of CIN2+ were similar for both age groups in WLWH (69.1% vs. 68.3%).

**Conclusions:**

Younger WLWH in Botswana had a significant burden of CIN2+. This finding further supports lowering the screening age for WLWH from 30 to 25.

## Background

Low- and middle-income countries (LMICs) carry the highest global burden of cervical cancer incidence and mortality [1]. Cervical cancer is the leading cause of cancer death in women in Southern Africa [2, 3]. While human papillomavirus (HPV) vaccination in young girls offers hope for a significant reduction in cervical cancer in future generations, effective cervical cancer screening services remain essential to reduce morbidity and mortality associated with cervical cancer in women across the globe [4].

Women living with the human immunodeficiency virus (WLWH) have a higher risk of developing pre-invasive cervical disease and cervical cancer [5–7]. Although progression rates from pre-invasive cervical disease to cervical cancer are unknown due to standard intervention for high-grade pre-cancer, cervical cancer is diagnosed at younger ages in WLWH compared to women without HIV [5, 8, 9]. Guidelines for high-income countries (HICs) recommend cervical cancer screening initiation at the early age of 21 [10–12]. Up until recently, guidelines for most LMICs recommended the initiation of cervical cancer screening at the age of 30 despite LMICs having the highest global prevalence of HIV in the reproductive-aged population [13, 14]. The 2021 WHO guidelines have a conditional recommendation based on low-certainty evidence for initiating screening at age 25 for WLWH [15], and call for more data. Further, many LMICs will not be able to change their guidelines immediately due to resource constraints.

Botswana has one of the highest HIV prevalences globally, at 25.1% in women aged 15–49 [16]. Botswana’s national guidelines prioritize screening in the 30 to 49 year-old age group with either cytology or visual inspection with acetic acid (VIA), regardless of HIV status. While practical, these guidelines may not adequately account for the high prevalence of HIV in Botswana and the higher risk of early cervical cancer progression. There is limited published data from Botswana on the prevalence of pre-invasive disease and the role of screening in younger women.

This study describes the association of age and HIV status with VIA positivity and high-grade cervical abnormalities. We aimed to determine how initiating cervical cancer screening at age 25 years, instead of 30 years, in WLWH would improve the identification of high-grade cervical pre-cancer without unduly increasing overtreatment of low-grade cervical pre-cancer. Data presented here could strengthen the evidence for the WHO recommendation on the target age group for cervical cancer screening in WLWH.

## Methods

### Study design and patient selection

We conducted a retrospective cross-sectional study based on the Botswana Ministry of Health and Wellness (MOHW) National Cervical Cancer Prevention Programme “see-and-treat” pilot programmatic database [17]. The evaluation included women screened between March 2009 and August 2015 with visual inspection after acetic acid (VIA) at Bontleng clinic, a primary care clinic in the capital city Gaborone providing HIV testing and anti-retroviral treatment (ART) for the district. Women with low-grade lesions were offered same-day treatment with cryotherapy for lesions that met eligibility criteria. Women with lesions ineligible for cryotherapy were referred to Princess Marina Hospital (PMH), a regional tertiary hospital located five kilometres away, for colposcopy and excisional procedure. Cervical cancer screening services were provided for WLWH as part of comprehensive HIV care. The services were only extended to women without HIV towards the end of the evaluation period. Screening services were offered free of charge to all Botswana citizens.

Screening services were linked to a physician-led referral colposcopy and loop electrosurgical excision procedure (LEEP) clinic at PMH. Through various channels, women came to screening services, including provider referral, self-referral following sensitization by written materials, and health education talks. Women were excluded from screening if they had previously had a hysterectomy, pelvic radiation for lower genital tract cancer, or a cervical cancer diagnosis. Screening for women who were menstruating heavily, pregnant, or had a persistent vaginal discharge was re-scheduled for after resolution of the condition.

### Cervical cancer screening procedures

All patients underwent a speculum examination of the cervix by a nurse who had participated in the Botswana MOHW VIA training program. The women were first assessed for lesions suspicious of cervical cancer (raised, ulcerative lesions with contact bleeding), and where present, women were referred to PMH for further evaluation. Visual assessment was performed after applying 5% acetic acid to the cervix using a cotton swab. The findings were categorized as normal, abnormal with a recommendation for cryotherapy, or abnormal with a recommendation for LEEP. Abnormal lesions were described as: (1) low-grade if they were well-defined and opaque acetowhite, or (2) high-grade if they were dense acetowhite or had abnormal vessels. Women with low-grade lesions covering less than three-quarters of the cervix, and not extending into the endocervical cancel were offered same-day treatment with cryotherapy; these women had no histopathology specimen collected. Women with abnormal lesions ineligible for cryotherapy based on appearance, size, or extension into the endocervical canal, were referred to the colposcopy/LEEP clinic at PMH, and evaluated by a specialist gynecologist or trained medical officers. The colposcopic appearance of lesions determined diagnostic and treatment decisions. Low-grade appearing lesions were treated with cautery after taking a biopsy; high-grade appearing lesions and those extending into the endocervical canal were treated with LEEP. Board-certified pathologists reviewed histopathology specimens at the National Health Laboratory (NHL), a government referral laboratory, offering services to the MOHW and private health facilities in Botswana. The pathologists were blinded to VIA findings. Histopathology results were classified according to the American Society of Colposcopy and Cervical Pathology (ASCCP) and the College of American Pathologists cervical intra-epithelial neoplasia (CIN) criteria. In brief, the specimens were recorded as no CIN, CIN graded one to three based on severity, or invasive cervical cancer (ICC).

### HIV procedures

Women with unknown HIV status at the time of screening or with documented HIV negative status more than six months prior were referred to an HIV testing center and requested to share their results. Throughout the study period, the Botswana National HIV program initiated anti-retroviral treatment (ART) at a CD4 count of ≤ 350 cells/µL.

### Data collection

All women undergoing VIA screening completed a questionnaire capturing a limited set of patient-level cervical cancer risk factors, including smoking, age of sexual debut, and parity. HIV status was recorded, and for WLWH, CD4 count at the time of HIV diagnosis and whether on ART at the time of screening was documented. VIA screening outcomes were recorded in the programmatic database. Histology results of women referred for colposcopy/LEEP were extracted from the NHL electronic medical record when available and entered into the programmatic database.

### Outcomes

The primary outcome was the association of VIA positivity and age, adjusting for cervical cancer risk factors. The secondary outcomes were the association of histopathologically confirmed high-grade abnormality and age, adjusting for cervical cancer risk factors; HIV-status association with VIA positivity and high-grade abnormality; and the proportions of VIA positivity and high-grade abnormality by both age and HIV status.

### Data analysis

The analyzed dataset included only women between the ages of 25 and 49. Patient records with missing data for VIA or histopathology that could not be corrected by cross-reference with primary records were excluded from the primary and secondary analysis, respectively. The sample size for the primary outcome was calculated using a 1-sided alpha of 0.05. To attain a 99% power, we assumed VIA positivity to be 30% in women aged 25 to 29 years and 20% in women aged 30 to 49 years based on previous findings [17]. The sample size required to detect a statistically significant difference in VIA-positivity between the two age groups was 2076 women (374 women aged 25 to 29 years and 1702 women aged 30 to 49 years).

The cervical cancer risk factors adjusted for included: HIV status, parity, smoking, and age of sexual debut. CD4 count and ART were included in the analysis of WLWH. Descriptive statistics for these variables are presented as median [interquartile range (IQR)] and proportions. Continuous variables were categorized into binary variables and compared using the chi-square test. Categorical variables included age groups of younger and older women (25 to 29 years; 30 to 49 years), age of sexual debut (≤ 18; > 18 years), parity (≤ 2; > 2), CD4 count (≤ 350 cells/µL; > 350 cells/µL), and histopathology results (benign or CIN 1 [≤ CIN1] for low-grade abnormalities; CIN2+ for high-grade abnormalities being CIN2/3 and ICC). Patterns of missing data were described for the study cohort using percentages.

Logistic regression models computed unadjusted and adjusted odds ratios (ORs) with 95% confidence intervals (CI). Only exposure variables with a *p*-value of less than 0.1 for unadjusted ORs were included in the adjusted regression models [18]. A *p*-value of less than 0.05 was considered to be statistically significant. We used Stata 14.0 (StataCorp LLC, College Station, Texas).

## Results

### Overall patient characteristics

**Fig. 1:**
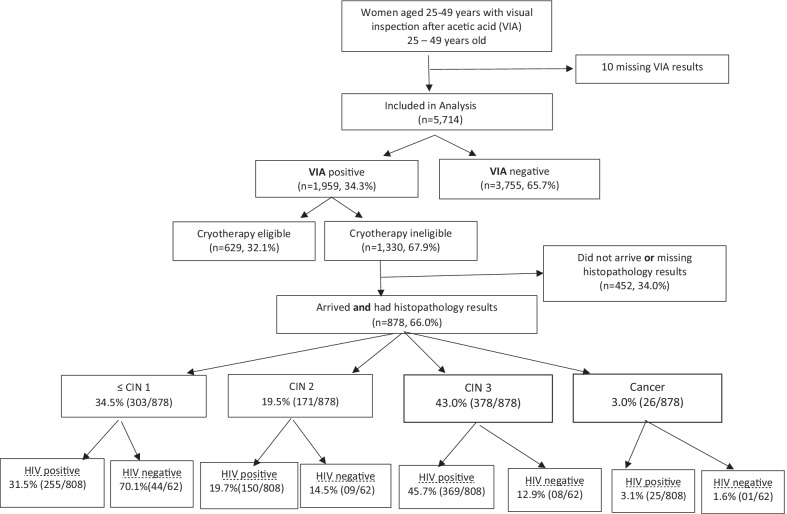
Study flow chart

The database included 5724 women aged 25 to 49 years screened with VIA between March 2009 and August 2015 (Fig. 1). Ten women had missing VIA data, leaving 5714 women for the VIA analysis. As shown in Table 1, the median age was 35 years [IQR 31–39], and 1029 (18%) were between 25 and 29 years of age. Smoking was reported by 285 (5%) of the women. The median age of sexual debut was 18 years [IQR 17–20], and the median parity was two [IQR 1–3]. HIV status was known in 5583 (98%), and 5026 (90%) of those with a known status were WLWH. Eight hundred and forty nine (86%) of the women aged 25 to 29 years and 4177 (91%) of the those aged 30 to 49 years were WLWH. Among the WLWH, the median CD4 count was 250 cells/µL [IQR 150–428], and 1628 (34.2%) were on ART. Missing data was ≤ 5% for all the variables except for CD4 count (11%, n = 551). The level of CD4 count missing data was similar for both age groups (10.5% for 25 to 29 year-olds versus 11.1% for 30 to 49 year-olds).

**Table 1:** Demographic and clinical characteristics of all study participants

Variable	All		Age 25–29 years	Age 30–49 years	*P*-value for χ^2^ test
	n (%)	Median [IQR]	n^a^ (%)	n^a^ (%)	
Age	5714	35 [31, 39]			
*Smoking*	5661		1018 (18.0)	4643 (82.0)	0.008^b^
Yes	285 (50.0)		68 (6.7)	217 (4.7)	
No	5376 (94.1)		950 (93.3)	4426 (95.3)	
Missing	53 (1.0)				
*Sexual debut*	5689	18 [17, 21]	1024 (18.0)	4665 (82.0)	0.02^b^
≤ 18	3150 (55.3)		533 (52.1)	2617 (56.1)	
> 18	2539 (44.4)		491 (47.9)	2048 (43.9)	
Missing	25 (0.4)				
*Parity*	5612	2 [1, 3]	1001 (17.8)	4611 (82.2)	< 0.001^b^
≤ 2	3223 (56.4)		815 (81.4)	2408 (52.2)	
> 2	2389 (41.8)		186 (18.6)	2203 (47.8)	
Missing	102 (1.8)				
*HIV status*	5583		989 (17.7)	4594 (82.3)	< 0.001^b^
Negative	557 (9.7)		140 (14.2)	417 (9.1)	
Positive	5026 (88.0)		849 (85.8)	4177 (90.9)	
Missing	131 (2.3)				
*Initial CD4 at HIV diagnosis*^c^	4475	250 [150, 428]	760 (16.9)	3715 (83.1)	< 0.001^b^
≤ 350	2308 (45.9)		304 (40.0)	2004 (53.9)	
> 350	2167 (43.1)		456 (60.0)	1711 (46.1)	
Missing	551 (11.0)				
*On ART at time of screening*^c^	4766		802 (16.8)	3964 (83.2)	< 0.001^b^
Yes	1628 (32.4)		346 (43.1)	1282 (32.3)	
No	3138 (62.4)		456 (56.9)	2682 (67.7)	
Missing	260 (5.2)				
*VIA results*	5714		1029 (18.0)	4685 (82.0)	< 0.001^b^
Positive	1959 (34.3)		428 (41.6)	1531 (32.7)	
Negative	3755 (65.7)		601 (58.4)	3154 (67.3)	

ART, anti-retroviral treatment; CIN, cervical intraepithelial neoplasia; IQR, inter-quartile range; VIA, visual inspection after acetic acid

^a^The number of women in levels of categorical variable may not add up to total “n” because missing category has been removed

^b^For p < 0.05

^c^For HIV positive patients only

### VIA-positivity

The overall VIA positivity of the study population was 34.3% (n = 1959). The proportion was higher in the 25 to 29 year-olds (41.5%, n = 428) than the 30 to 49 year-olds (32.7%, n = 1531). The WLWH had a higher VIA positivity rate (35.9%, n = 1841) than women without HIV (24.1%, n = 141) (Table 2).

**Table 2:** Study participants’ characteristics with bivariate and multivariable odds ratio for VIA positivity

Variable	All n (%)	VIA positive n^a^ (%)	VIA negative n^a^ (%)	VIA positivity BVA Odds Ratios (95% CI)	*P*-value	VIA Positivity MVA Odds Ratios (95% CI)	*P*-value for χ^2^ test
*Age Group*	5714	1959 (34.3)	3755 (65.7)				
25–29 years	1029 (18.0)	428 (41.6)	601 (58.4)	1.47 (1.28, 1.69)	< 0.001^b^	1.48 (1.28, 1.72)	< 0.001^b^
30–49 years	4685 (82.0)	1531 (32.7)	3154 (67.3)	Ref			
*Smoker*	5661	1937 (34.2)	3724 (65.8)				
No	5376 (94.1)	1825 (33.9)	3551 (66.1)	Ref			
Yes	285 (5.0)	112 (39.3)	173 (60.7)	1.27 (1.00, 1.62)	0.053	1.14 (0.89, 1.46)	0.31
Missing	53 (0.9)						
*Age sexual debut*	5689	1950 (34.3)	3739 (65.7)				
≤ 18	3150 (55.1)	1075 (34.1)	2075 (65.9)	0.99 (0.88, 1.10)	0.79	N/A	N/A
> 18	2539 (44.4)	875 (34.5)	1664 (65.5)	Ref			
Missing	25 (0.5)						
*Parity*	5612	1924 (34.3)	3688 (65.7)				
≤ 2	3223 (56.40)	1151 (35.7)	2072 (64.3)	Ref			
> 2	2389 (41.81)	773 (32.4)	1616 (67.6)	0.86 (0.77, 0.96)	0.009^b^	0.88 (0.79, 0.99)	0.04^b^
Missing	102 (1.79)						
*HIV status*	5583	1941 (34.8)	3642 (65.2)				
Negative	557 (9.74)	134 (24.1)	423 (75.9)	Ref			
Positive	5026 (87.96)	1807 (35.9)	3219 (64.1)	1.77 (1.45, 2.17)	< 0.001^b^	1.85 (1.51, 2.28)	< 0.001^b^
Missing	131 (2.30)						
*CD4 Count at HIV*	4475	1620 (36.2)	2855 (63.8)				
Diagnosis^c^	2308 (45.9)	809 (35.1)	1499 (64.9)	0.90 (0.80, 1.02)	0.10	0.96 (0.83, 1.10)	0.52
≤ 350	2167 (43.1)	811 (37.4)	1356 (62.6)	Ref			
> 350	551 (11.0)						
Missing							
*ART at time of screening* ^c^	4766	1710 (35.9)	3056 (64.1)				
No	3138 (62.4)	1096 (34.9)	2047 (65.1)	0.89 (0.78, 1.00)	0.06	0.91 (0.78, 1.05)	0.19
Yes	1628 (32.4)	614 (37.7)	1014 (62.3)	Ref			
Missing	260 (5.2)						

ART, anti-retroviral therapy; BVA, bivariate analysis; MVA, multivariate analysis; VIA, visual inspection after acetic acid

^a^The number of women in levels of categorical variable may not add up to total “n” because missing category has been removed

^b^For *P* < 0.05

^c^For HIV positive patients only

In multivariate analyses, VIA positivity was more likely in 25 to 29 year-olds than in 30 to 49 year-olds (OR 1.48, CI 1.28, 1.72), and in WLWH compared to women without HIV (OR 1.85, CI 1.51, 2.28). Among WLWH, VIA positivity was not affected by CD4 count (OR 0.96, CI 0.83, 1.10) or by ART (OR 0.91, CI 0.78, 1.05) (Table 2).

### High-grade abnormality

The majority of the VIA-positive lesions were ineligible for treatment with cryotherapy (68%, n = 1330); this was similar for both the 25 to 29 year-olds and the 30 to 49 year-olds (67.1% vs. 68.1%, respectively). Of the 1330 women referred to colposcopy/LEEP, 878 (66%) attended and had recorded histopathology results (58.5% for 25 to 29 year-olds, and 68.1% for 30–49 year-olds). The rates of ≤ CIN1, CIN2, CIN3 and ICC among women with histopathology results were 33.4%, 19.5%, 43.0%, and 3.0%, respectively. Although the CIN 2/3 rates were similar for both age groups, all cancers were in the 30 to 49 age group except for one case recorded in the 25 to 29 age group of WLWH (Fig. 1, Table 3).

**Table 3:** VIA and histological outcomes by age group and HIV status

	All participants		HIV positive^a^		HIV negative^b^	
	25–29 Age group	30–49 Age group	25–29 Age group	30–49 Age group	25–29 Age group	30–49 age group
Number Screened with VIA	N = 1029	N = 4685	n = 849	N = 4177	N = 140	N = 417
VIA* outcomes*						
*VIA results*						
Negative	601 (58.4%)	3154 (67.3%)	472 (55.6%)	2747 (65.8%)	95 (67.9%)	328 (78.7%)
Positive	428 (41.6%)	1531 (32.7%)	377 (44.4%)	1430 (34.2%)	45 (32.1%)	89 (21.3%)
Eligible for cryotherapy	141 (32.9%)	488 (31.9%)	121 (32%)	477 (33.4%)	18 (40%)	34 (38.2%)
Not eligible for cryotherapy	287 (67.1%)	1043 (68.1%)	256 (68%)	953 (66.6%)	27 (60%)	55 (61.8%

Not eligible for cryotherapy, arrived at colposcopy with histology results	N = 168	N = 710	n = 149	n = 659	N = 16	N = 46
*Histology outcomes*						
≤ CIN 1	56 (33.4%)	247 (34.8%)	46 (30.9%)	209 (31.7%)	8 (50%)	36 (78.2%)
CIN2	36 (21.4%)	135 (19.0%)	31 (20.8%)	128 (19.4%)	5 (31.3%)	4 (8.7%)
CIN3	75 (44.6%)	303 (42.7%)	71 (47.6%)	298 (45.2%)	3 (18.7%)	5 (10.9%)
Cervical cancer	1 (0.6%)	25 (3.5%)	1 (0.7%)	24 (3.7%)	0 (%)	1 (2.2%)

CIN, cervical intraepithelial neoplasia; VIA, visual inspection after acetic acid

Figures for ^a^ and ^b^ do not add up to 5714 due to missing HIV status in 131 records

In multivariate analyses, CIN2 + was associated with a positive HIV status (aOR 6.12, CI 3.39, 11.10), but not with age (OR 1.07, CI 0.75–1.52 for 25 to 29 year-olds compared to 30 to 49 year-olds). In WLWH, neither CD4 count nor ART was associated with CIN2+ (Table 4).

**Table 4:** All Study participants’ characteristics with bivariate and multivariable odds ratio for CIN2 +

Variable	All n (%)	≥ CIN2+ n^a^ (%)	≤ CIN1 n^a^ (%)	CIN2+ BVA Odds Ratios (95% CI)	*P*-value	CIN2+ MVA Odds Ratios (95% CI)	*P*-value for χ^2^ test
*Age Group*	878	575 (65.5)	303 (34.5)				
25–29 years	168 (19.1)	112 (66.7)	56 (33.3)	1.07 (0.75, 1.52)	0.72	N/A	N/A
30–49 years	710 (80.9)	463 (65.2)	247 (34.8)	Ref			
*Smoker*	867	566 (65.3)	301 (34.7)				
No	817 (93.1)	530 (64.9)	287 (35.1)	Ref			
Yes	50 (5.7)	36 (72.0)	14 (28.0)	1.38 (0.74, 2.62)	0.30	N/A	N/A
Missing	11 (1.2)						
*Age Sexual debut*	873	572 (65.5)	301 (34.5)				
≤ 18	505 (57.5)	338 (66.9)	167 (33.1)	1.16 (0.87, 1.54)	0.31	N/A	N/A
> 18	368 (41.9)	234 (63.6)	134 (36.4)	Ref			
Missing	5 (0.6)						
*Parity*	861	566 (65.7)	295 (34.3)				
≤ 2	517 (58.9)	328 (63.4)	189 (36.6)	Ref			
> 2	344 (39.2)	238 (69.2%)	106 (30.8)	1.29 (0.97, 1.73)	0.08	1.30 (0.96, 1.75)	0.09
Missing	17 (2.0)						
*HIV Status*	870	571 (65.6)	299 (34.4)				
Negative	62 (7.1)	18 (29.0)	44 (71.0)	Ref			
Positive	808 (92.0)	553 (68.4)	255 (31.6)	5.30 (2.96, 9.49)	< 0.001^b^	6.12 (3.39, 11.10)	< 0.001^b^
Missing	8 (0.9)						
*CD4 Count at HIV*	701	494 (70.5)	207 (29.5)				
Diagnosis^c^	336 (41.6)	234 (69.6)	102 (30.4)	0.93 (0.67, 1.28)	0.65	N/A	N/A
≤ 350	365 (45.2)		105 (28.8)	Ref			
> 350	107 (12.2)						
Missing		260 (71.2					
*ART at time of screening* ^c^	773	527 (68.2)	246 (31.8)				
No	455 (56.3)	315 (69.2)	140 (30.8)	1.13 (0.83, 1.53)	0.45	N/A	N/A
Yes	318 (39.4)	212 (66.7)	106 (33.3)	Ref			
Missing	35 (4.3)						

ART, anti-retroviral therapy; BVA, bivariate analysis; MVA, multivariate analysis; VIA, visual inspection after acetic acid

^a^The number of women in levels of categorical variable may not add up to total “n” because missing category has been removed

^b^For *P* < 0.05

^c^For HIV positive patients only

### VIA-positivity and high-grade abnormality by age and HIV-status

In WLWH, the 25 to 29 year-olds were more likely to be VIA positive than the 30 to 49 year-olds (44.4%, n = 377 vs. 34.2%, n = 1430, respectively). We observed a similar pattern for women without HIV (32.1%, n = 45 for 25 to 29 year-olds, compared to 21.3%, n = 89 for 49 year-olds). Among women with histopathology results, the rate of CIN2 + in WLWH was 68.4% (69.1% for the 25 to 29 year-olds and 68.3% for the 30 to 49 year-olds), and the rate of CIN2 + in women without HIV was 29.0% (50% for 25 to 29 year-olds and 21.8% for 30 to 49 year-olds) (Table 3).

## Discussion

WLWH aged 25 to 29 years attending routine cervical cancer screening in our national program had the same odds of having high-grade cervical pre-cancer as women aged 30 to 49 years. Prior research has indicated a link between younger age and cervical cancer among WLWH [5, 8, 9]. Our findings confirm the presence of a significant level of cervical cancer precursors requiring intervention in women as young as 25 years, particularly in WLWH, thus supporting the 2021 WHO recommendation to lower the age of initiation of cervical cancer screening from 30 to 25 years in WLWH.

A concern about lowering the cervical cancer screening age has been that clinically insignificant lesions from transient HPV infections would be intervened upon unnecessarily, resulting in overtreatment of young women [19, 20]. Although women in this cohort aged 25 to 29 years had higher rates of VIA positivity than women aged 30 to 49 years, similar proportions were referred for an excisional procedure. The histopathology results indicate that the proportions of CIN2+ detected and appropriately treated were similar for the two age groups in WLWH. If overtreatment did occur, it would primarily have occurred in the group of women treated with cryotherapy, a treatment that ultimately has minimal side effects [21]. As expected, the proportion of CIN2+ was more than three times lower in women without HIV than in WLWH. In the group without HIV, women aged 25 to 29 were twice as likely to have CIN2+ as women aged 30 to 49, a finding that is not in keeping with the general literature. However, it is difficult to draw any conclusion from this finding due to the small number of the women without HIV in the cohort.

We had expected to find a correlation between patient age and high-grade pre-cancer because older women would have had a longer time to progress from HPV infection to cervical pre-cancer without opportunities for intervention [22]. However, our data do not support this hypothesis. Instead, younger women overall and younger WLWH had a similar proportion of CIN2+ compared to older women. Data is limited on HPV progression to cervical pre-cancer and cancer in women aged 20 to 29 years. Adolescent WLWH are more likely to have HPV co-infections and coexisting abnormalities, albeit low-grade, relative to those without HIV [23]. Our finding of high rates of high-grade pre-cancer in 25 to 29 year-old WLWH further supports an accelerated timeframe of progression from HPV infection to high-grade pre-cancer in young WLWH.

Our analysis has limitations. We utilized a programmatic database from a screening programme originally designed to serve only WLWH. Services were only later offered to women without HIV; hence there is a much lower number of women without HIV in this cohort than the general population. Therefore, the findings may not be fully generalizable to all females aged 25–49 years in Botswana. Further, limited patient-level demographic and risk factor data were collected, and not all variables had complete data. For instance, CD4 count had 11% missing data; however, this was similar for both age groups, and we doubt that it would have had a significant effect on the outcomes. Other key HIV-related variables, including viral load and timing of HIV treatment, were not collected. Therefore, the full extent of the immune status of WLWH could not be assessed. Although the rates of VIA positivity and cryotherapy ineligibility were high, similar rates have been observed in other high HIV burden areas and could be related to low rates of prior screening in the population [24]. The high VIA positivity rates in this cohort could also be related to non-HPV-associated cervicitis. High rates of cervicitis have been shown to affect the accuracy of VIA in women in the 30 to 49 age group in a similar population [25]. However, while cervicitis may affect VIA positivity rates in both younger and older age groups, the higher cervicitis rate in the younger group may have accounted for a greater increase in VIA than in the older age group. Over 30% of the women were eligible for cryotherapy without biopsy, which means some CIN2+ lesions might have been treated with ablative treatment without documented histopathology results leading to the underestimation of the CIN2+ rate. Finally, a third of the women with lesions ineligible for cryotherapy did not comply with their referral for colposcopy, and documentation of colposcopy/LEEP referral appointment attendance was not fully recorded. Thus, histopathology results may not represent the entire cohort of women who had or should have had colposcopic evaluation.

## Conclusions

Despite the limitations of this study, we present new evidence of the significant burden of CIN2+ in younger WLWH in Botswana. Until the population-level effects of HPV vaccination and universal ART to improve overall immune competence in WLWH are realized [26], the reduction in cervical cancer in LMICs will depend on effective, comprehensive screening programs for WLWH. This additional evidence further supports the current WHO conditional recommendation for initiating screening at age 25.

## Data Availability

The dataset generated during the current study is provided.

## Abbreviations

ART: Anti-retroviral treatment
CI: Confidence interval
CIN: Cervical intraepithelial neoplasia
HIC: High-income country
HIV: Human immunodeficiency virus
HPV: Human papillomavirus
HRDC: Health research and development committee
LEEP: Loop electro-surgical excision procedure
LMIC: Low-middle income county
IQR: Interquartile range
MOHW: Ministry of health and wellness
NHL: National health laboratory
OR: Odds ratio
PMH: Princess Marina hospital
VIA: Visual inspection after acetic acid
WHO: World Health Organization
WLHV: Women living with HIV
